# Daily physical activity of Brazilian carriers of arterial hypertension: a transversal analysis

**Published:** 2017-06-30

**Authors:** Dartel Ferrari de Lima, Lohran Anguera Lima, Olinda do Carmo Luiz

**Affiliations:** 1 Department of Physical Education, Universidade Estadual do Oeste do Paraná, Marechal Cândido Rondon, Brazil.; 2 Department of Orthopedics and Traumatology, Santa Casa de Misericórdia of São Paulo, São Paulo, Brazil.; 3 Department of Preventive Medicine, College of Medicine, Universidade de São Paulo, São Paulo, Brazil.

**Keywords:** Health promotion, physical activity, arterial hypertension, Preventive Medicine, epidemiology, sedentary lifestyle

## Abstract

**Objective:**

To describe the profile of the practice of physical activity in the daily life of Brazilian adults with arterial hypertension and to analyze whether the practice performed complies with the recommendations of the World Health Organization.

**Methods:**

Cross-sectional data were obtained from the Surveillance System of Risk Factors and Protection for Chronic Noncommunicable Diseases of 2014, involving 40,853 adults aged 18 years and over in all Brazilian capitals, interviewed by telephone survey.

**Results:**

Walking, soccer and water aerobics were the main modalities of exercise and sport practiced. The weekly volume of effort led 35% of practitioners to reach the recommended goal of the World Health Organization. The low weekly frequency of activities stood out among hypertensives who did not reach the goal.

**Conclusion:**

Health services should clarify the need for regularity of physical activity for hypertensive individuals to benefit substantially.

## Resumen

**Objetivo:**

Describir el perfil de la actividad física diaria en brasileños adultos con hipertensión arterial y analizar si la actividad practicada cumple con las recomendaciones de la Organización Mundial de la Salud.

**Métodos:**

Los datos transversales se obtuvieron del Sistema de Vigilancia de Factores de Riesgo y Protección para Enfermedades Crónicas no Transmisibles de 2014, donde cerca de 40,853 adultos mayores de 18 años fueron entrevistados por la encuesta telefónica.

**Resultados:**

La caminata, fútbol y Hidrogimnasia fueron las principales formas de ejercicio y el deporte practicado. Volumen semanal del esfuerzo llevó el 35% de los hipertensos para alcanzar el objetivo AF recomendado por la Organización Mundial de la Salud. La frecuencia reducida de actividades semanales se destacó entre los hipertensos que no alcanzaron el objetivo.

**Conclusión:**

Los servicios de salud deben hacer explícita la necesidad de la regularidad de la actividad física a hipertensos para su beneficio sustancial.

## Introduction

Worldwide, it has been recorded that approximately one-third of hypertensive patients are unaware of their condition, consequently, there is a risk increase regarding myocardial infarction, stroke and kidney diseases. Hypertension also accounts for almost 9.4 million deaths and 10% cost in health. It is estimated that, in 2025, hypertension will affect nearly 1.6 billion people of both sexes ^1^. In Brazil, hypertension has reached 30% of the adult population, 5% in children and adolescents and 50% in elderly people ^2^.

According to a study that was carried out in 190 countries from 2001 to 2011, Brazil has the sixth largest rate of hypertensive people in the world (552/100,000) with a 13.2% increase in this disease during this trial ^3^. There is a positive and promising aspect based on this scenario that deserves to be highlighted, which is the fact that hypertension can be well controlled with the use of antihypertensive drugs, plus healthy living habits as a decision-making. Regular physical activities (PA) have been recognized as an antihypertensive effect as well as a complementary therapeutic attitude.

Fagard ^4^ carried out a meta-analysis that showed some significant decrease in systolic and diastolic blood pressure (3.4 and 2.4 mmHg, respectively, *p* <0.001), caused by aerobic training performed 3 to 5 times a week for about 30 to 60 min. This result was later corroborated by other studies ^5^ ^-^ ^7^.

In Brazil, a population study based on data from the National System for Monitoring Hypertension and its risk factors analyzed the PA level of 9,038 in self-reported hypertensive participants, 60 years old or more. The answers showed a high prevalence of insufficient AP during leisure time (88%; 95% CI: 86-89) ^8^.

Another representative study concerning Brazilian population analyzed 11,453 hypertensive patients of 18 years old or more and showed 59.5% (95% CI: 57.8-61.0) of inactive/sedentary patients, 10.1% (95% CI: 8.8 - 12.2) of insufficient actives and 30.4% (95% CI: 26.8-32.8) of sufficient actives (*p*= 0.001) ^9^.

Currently, several organizations have recommended PA to improve health of hypertensive people. At first glance, there seems to be ambiguous in recommending PA along the week, however, organizations agree on the need of achieving a minimum dose required to result in substantial effects. For example, the American College of Sports Medicine recommends exercises of mild to moderate intensity for about 20-40 min a day, which should be done continuously or at intervals and with minimum frequency of three non-consecutive days a week ^10^.

The Brazilian Ministry of Health recommends 3 to 5 AP times a week, during at least 30 min a day, whose intensity depends on the individual's physiological ability ^11^.

The World Health Organization (WHO) accompanied by the United States Department of Health and supported by clear evidence that health benefits of hypertensive individuals occur despite which physical exercise someone works out and by different effort intensity. There is an aim of working out for 150 min a week on aerobic activities of moderate intensity or 75 min of vigorous intensity or an equivalent combination of both, preferably, but not necessarily distributed throughout the week. Although, it should be highlighted the exercise and frequency of activity based on each individual's ability and health limitations ^12^ ^,^ ^13^.

In this perspective, this study proposed to describe the participation profile of hypertensive Brazilian adults PA, who live in some Brazilian capitals and in the Federal District as well as analyze their workout intensity/ how intense were their physical exercises according to WHO recommendations.

## Material and Methods

Cross-sectional data from the Surveillance of Risk Factors and Protection for Non-Communicable Chronic Diseases system were examined by telephone interview (Vigitel) in Brazil based on 2014 year, whose own methodology can be accessed in a previous publication ^14^. Since 2006, the system interviews have occurred annually and are registered by telephone interview with adults from 26 Brazilian capitals and the Federal District. They were 18 years old or more and had landlines. Using strategic monitoring reasons, some survey issues may be added or withdrawn. And, issues regarding PA participation have not changed over time. In 2014, 62,786 lines were eligible, resulting in 40,853 valid interviews, so, there was an average success rate of 65.2%. The interviews lasted 9.5 minutes in average and were recorded by an electronic questionnaire about demographic, socioeconomic and behavioral characteristics of the individuals as well as questions about daily PA practice.

Thus, in order to adjust the adult population of each city, a final weight was assigned to each individual interviewed by Vigitel, named as post-stratified weight. This aimed at correcting the representation of socio-demographic strata that could occur due to the non-universal coverage of the fixed-line network ^15^.

### Variables

This study considered as hypertensive the participant who answered yes to this question: has any doctor ever told you that you have high blood pressure? For estimates of PA level, it was considered the sum of weekly frequency and the duration of activities practiced in free time or on his way to the work and/or school. So, the following questions were used: "have you practiced any kind of physical exercise or sport during the last three months? Yes/No/Which?" And the question: "do you do any walking or cycling when going to or coming from your work/ school? Yes/No". The effort intensity for each kind of physical exercise or sports followed the classification established by Vigitel in 2014 edition.

Thus, walking, treadmill walk, water aerobics, general gymnastics, swimming, cycling and volleyball were classified as moderate PA practices. Running, treadmill running, bodybuilding, aerobic exercise, martial arts/fights, soccer, basketball and tennis were classified as vigorous PA practices. The daily effort duration was determined by asking: "on the day you exercise or practice a sport, how long does this activity last?" "How much time do you spend to go back and forth on this route (on foot or by bicycle)?" The analysis was categorized into six groups: less than 10 min; 10 to 19 min; 20 to 29 min; 30 to 39 min; 45 to 59 min; And equal to or greater than 60 min.

Therefore, an activity that lasts less than 10 min was not considered for calculation purposes ^12^. The weekly frequency was determined by the number of active days along the week by asking: "how many days in a week do you usually exercise or practice a sport?" For the analysis, the weekly frequency was categorized into four groups: daily, 5 to 6 days, 3 to 4 days and 1 to 2 days a week.

The analysis of PA participation considered only the last three months prior to the interview. Originally, the Vigitel survey assessed PA separately in four domains: PA during leisure time; Occupational PA (work); Residential PA (domestic cleaning); And PA on moving from home to work or school (on foot or by bicycle). In this study, only information concerning PA leisure and active displacement were used to define the participants' PA level. The activities that were done at home or work were not considered because the Vigitel questionnaire did not predict duration or weekly frequency in those activities.

Brazilian regions separation attended the grouping of the Federation units. Currently, in Brazil, there are five official regions: Central-West Region with three states (Goiás, Mato Grosso and Mato Grosso do Sul) and the Federal District; Northeastern Region with nine states (Alagoas, Bahia, Ceará, Maranhão, Paraíba, Pernambuco, Piauí, Rio Grande do Norte and Sergipe); Northern Region with seven states (Acre, Amapá, Amazonas, Pará, Rondônia, Roraima and Tocantins); Southeastern Region with four States (Espírito Santo, Minas Gerais, Rio de Janeiro and São Paulo); Southern Region with three states (Paraná, Rio Grande do Sul and Santa Catarina).

The determination of PA level applied WHO global parameters of 2010, which considered adequate a moderate practice that lasts at least 150 minutes or as vigorous PA, the one that last 75 minutes a week, regardless of its weekly frequency ^12^. Initially, the participants were grouped into four categories: 1) inactive: denied practicing PA during leisure time and his active moving to work or school; 2) insufficient active: performed PA below the WHO recommendations; 3) active: the one who reached PA goal recommended by WHO; And 4) very active: the one who exceeded the WHO recommendations. A second grouping was carried out considering two other categories: The category 'meets the WHO recommended goal', denominated Group A, was composed of the sum of active and very active participants, and the category that 'does not meet the goal recommended by the WHO, named as Group B, was composed of the sum of the inactive and insufficient active participants.

PA prevalence of hypertensive participants and their respective 95% confidence intervals were presented according to individual PA level, by region and by exercise and/or sport modality. PA characteristics in hypertensive population were described in order to try to compare them with characteristics of the population in general. The possible differences in PA prevalence according to the presence or absence of a disease were evaluated using the chi-square (χ^2)^ of Pearson test. The results with *p* lower than 0.05 were considered with statistically significant difference. And Stata ^®^ 11.0 and Microsoft(r) Excel 10.0 programs were used to process statistical data and analyses.

### Ethical aspects

The present study applied secondary data collected by Vigitel system. Vigitel project was approved by the National Commission of Ethics in Research for Human Beings of the Ministry of Health (CONEP-Legal Opinion 355,590 of 06/26/2013-CAAE: 16202813.2.0000.0008).

Free and informed consent was obtained orally at the time of telephone contact with the interviewees and anonymity was guaranteed for participants. In the database, made publicly available by the Ministry of Health at: http://svs.aids.gov.br/bases_vigitel_viva/vigitel.php, the participants are numbered sequentially, without any possibility to be identified, according to resolution 466/2012 of the National Health Council ^15^.

## Results

Cross-sectional data from Vigitel of 2014 were analyzed, with 40,853 interviewees, whose mean age was 48.3 ± 17.6 years old, 15,521 men with mean age of 45.9 ± 17.5 years old and 25,332 women whose mean age was 49.7 ± 17.5 years old. The majority declared to be white (44.9%) or brunette (40.9%). Only 1.6% of them declared to be indigenous and 9.8% declared themselves as black. Brazilian regions with the highest number of states showed higher gross participation, although post-stratification weights were assigned to each individual to get closer to sociodemographic composition of the sample to that one observed in the Demographic Census in each city.

A total of 1,405 participants (3.4%) were excluded, although they have reported to have some kind of PA practice, and they did not report their weekly frequency or duration of exercise. Thus, the calculation base considered 39,448 participants. Among 23.5% (95% CI: 21.6-25.4), who confirmed the diagnosis of hypertension, there were more women 25.8% (95% CI: 22.2-27.6) than men 20.3% (95% CI: 18.4-22.9) (*p*= 0.001). Palmas city had the lowest prevalence of hypertension (15.2%) and Porto Alegre, the highest one (29.2%). The distribution of hypertensive people was unequal according to schooling, with 38.1% (95% CI: 36.6-40.8) in people with up to 8 years of schooling; 19.3% (95% CI: 17.5-21.7) between 9 to 11 years and 14.6% (95% CI: 11.9-16.6) over 12 years of schooling (*p*= 0.001).

Brunet people had the lowest prevalence of hypertension (4.7%), while Asian descendants showed the highest answer (10.4%) (*p*= 0.01). In both sexes, hypertension prevalence was higher with advancing age. It affected approximately 4.6% of 18-24 year-old people and 59.9% of 65 years of age or older.

Table 1 shows the distribution of hypertensive patients according to PA level. It can be observed that more than a half (59.5%) were inactive and only 34.4% showed adequate PA. 

**Table 1:** Percentage distribution^*^ of the hypertensive adults (aged ≥18 years), by sex, according to the physical activity (PA) level determined by WHO^§^ recommendations (2010), in the Brazilian capitals and the Federal District, 2014.

	Total		Men		Women	
Level of PA	%^#^	95% CI	%^#^	95% CI	%^#^	95% CI
Inactive	59.3	55.8-59.2	54.3	50.6-56.4	64.3	62.1-6.9
Insufficient active	10.7	8.8-12.2	13.5	11.3-14.3	7.8	6.6-9.1
Active	8.8	7.1-9.5	7.9	6.0-9.3	7.8	6.3-8.6
Very active	21.2	19.7-23.3	24.3	22.8-6.3	20.1	18.9-2.9
Group A^**^	34.4	31.4-38.1	39.8	35.3-43.9	31.3	28.6-5.9
Group B^***^	65.6	61.9-69.4	60.2	56.1-65.2	68.7	64.0-62.1

^§^ World Health Organization.

* Percentage weighted to adjust the Vigitel-2014 sample distribution to the population distribution projected for the 2014 year.

** Reach the WHO recommendations goals (2010) - Sum of active and very active.

*** Don't reach the WHO recommendations goals (2010) - Sum of inactive and insufficient active.

^#^Pearson's Chi-square test <0.005.

95% CI - 95% Confidence interval.

Table 2 presents the prevalence of hypertensive patients and the percentage that reached WHO recommendation regarding physical activities, by Brazilian regions. Among men, hypertensive individuals were more active in the Central Western Region (44.1%; 95% CI: 24.0-53.0) and less active in the Southern Region (36.6%; 95% CI: 26.0-46.0). Among women, the hypertensive ones were more active in the Central Western Region (36.6%; 95% CI: 28.0-44.0) and less active in the Northern Region (27.3%; 95% CI: 19.0-36.0).

**Table 2:** Percentage distribution* of the hypertensive adults (aged ≥ 18 years), by sex, that achieved the WHO^§^ physical activity (PA) recommendation goals (2010), in the Brazilian regions, 2014.

Regions	Prev ^§^ %	Goal achieved^&^	
		Men (95% CI)	Women (95% CI)
North	19.4	36.6 (26.0-46.0)	27.3 (19.0-36.0)
Northeast	24.3	41.9 (31.0-48.1)	30.7 (22.0-38.0)
Midwest	24.7	44.1 (24.0-53.0)	36.6 (28.0-44.0)
Southeast	26.1	36.7 (26.0-46.0)	30.2 (26.0-38.0)
South	25.0	38.4 (29.0-49.0)	36.1 (29.0-43.5)

^& χ2= <0.001^

^§^ Prev= Prevalence of self-reported arterial hypertension

*Percentage weighted to adjust the Vigitel-2014 sample distribution to the population distribution projected for the 2014 year

CI= Confidence interval.

Table 3 presents the main modality of physical exercise or sport practiced by hypertensive individuals. Walking was the main activity, and it was more practiced by individuals of Group A with 67.9% (95% CI: 64.8-70.8) when compared to those ones from Group B (44.8%; 95% CI: 39.9-49.7). Soccer preference was predominantly male, especially for Group B components with 41.6% (95% CI: 34.1-49.1). The sum of the set of sports practiced in sports courts contributed with only 1% total of choices.

**Table 3:** Percentage^*^ of the adult population arterial hypertensive self - reported and physical activity (PA) level, by sex, according to the physical exercise type or sport, in the Brazilian capitals and Federal District, 2014.

	Group A^**^						Group B^***^					
Modalities	Total %	(95% CI)	Man %	(95% CI)	Woman %	(95% CI)	Total %	(95% CI)	Man %	(95% CI)	Woman %	(95% CI)
1 = walking	67.9	64.8-70.8	60.4	55.3-65.4	73.7	70.4-7.5	44.8	39.9-49.7	31.2	25.1-37.3	59.9	53.0-66.7
2 = treadmill walk	1.9	1.2-2.6	2.6	1.4-3.8	1.4	0.6-2.2	1.6	0.5-2.7	1.8	0.01-3.5	1.5	0,2-2.8
3 = run	2.7	1.7-3.7	3.9	1.9-5.8	1.8	0.9-2.6	2.0	0.8-3.1	2.3	0.6-4.0	1.5	0.08-3,2
4 = treadmill run	0.3	0.05-0.6	0.6	0.01-1.2	0.1	0.04-1.3	0,4	0.1-0.8	0.6	0.3-1.6	0.04	0.05-0.1
5 = bodybuilding	6.3	4.6-7.8	7,5	4.8-10.2	5,2	3.4-7.1	2.9	0.7-5.1	3.7	0.1-7.5	2.0	0,2-3.8
6 = aerobic gymnastics	2.0	1.0-3.0	0.7	0.02-1.4	3.0	1.3-4.7	1.3	0.6-1.9	0.6	0.05-1.3	1.9	0.8-3.1
7 = waterobics	3.7	2.5-4.8	1.1	0.3-2.1	5.7	3.8-7.6	5.4	3.1-7.7	1.5	0.2-2.8	9.8	5.3-14.3
8 = general gymnastics	2.6	1.8-3.3	1.3	0.5-2.0	3.6	2.3-4.9	4.6	3.0-6.2	2.7	0.9-4.5	6.7	4.1-9.4
9 = swimming	0.9	0.4-1.4	1.1	0.3-1.9	0.7	0.005-1.4	2.3	1.0-3.6	2.6	0.6-4.5	2.0	0.3-3.8
10 = martial arts	0.3	0.02-0.5	0.6	0.03-1.2	0.2	0.001-0.05	0.3	0.03-0.6	0.3	0.1-0.8	0.2	0.2-0.6
11 = cycling	3.2	2.4-4.1	4.4	3.0-5.8	2.3	1.2-3.3	5.0	2.4-7.7	5.4	2.4-8.4	5.0	0.3-9.1
12 = football	4.7	3.1-6.3	10.5	7.0-13.9	0.05	0.004-0.1	22.2	17.4-27.0	41.6	34.1-49.1	0.6	0.2-1.2
13 = basketball	0.1	0.01-0.3	0.2	0.03-0.5	0.01	0.007-0.02	0.005	0.005-0.02	0.01	0.001-0.03	-	-
14 = volleyball	0.2	0.05-0.5	0.4	0.02-1.1	0.06	0.005-0.01	1.0	0.2-2.3	0.6	0.07-1.0	1.5	1.1-4.1
15 = tennis	0.1	0.01-0.02	0.3	0.01-0.5	0.08	0.01-0.1	0.3	0.04-0.6	0.4	0.02-0.8	0.2	0.1-0.5
16 = other	3.1	1.8-4.4	4.4	1.9-6.8	2.1	0.8-3.4	5.9	3.8-8.0	l4.7	1.5-7.9	7.2	5.6-10.0

* Percentage weighted to adjust the Vigitel-2014 sample distribution to the population distribution projected for the 2014 year.

** Reach the WHO recommendations goals (2010) - Sum of active and very active.

*** Don't reach the WHO recommendations goals (2010) - Sum of inactive and insufficient active.

95% CI - 95% Confidence interval.

Table 4 shows the distribution of weekly frequency and daily activities duration. Most men (39.5%; 95% CI: 34.4-44.5) and women (43.1%; 95% CI: 38.9-47.3) participated of PA from 3 to 4 days a week. Only 5.5% (95% CI: 3.2-7.7) and 1.1% (95% CI: 0.2-1.9) of the studied women had a minimum frequency of 1 to 2 days a week. More than half of hypertensive individuals exercised 60 min or more on active days and approximately 90% exercised 30 minutes or more. 

**Table 4:** Percentage distribution^*^ of the hypertensive adults (aged ≥ 18 years), by sex, according to the weekly frequency and daily duration of the physical activity (PA) participation, in the Brazilian capitals and Federal District, 2014.

Group A^**^	Men (%)	95% CI	Women (%)	95% CI
Weekly frequency (days)				
Daily	29.8	(24.2-35.4)	22.7	(18.8-26.6)
5-6	25.2	(20.4-30.0)	33.1	(29.1-37.1)
3-4	39.5	(34.4-44.5)	43.1	(38.9-47.3)
1-2	5.5	(3.2-7.7)	1.1	(0.2-1.9)
Duration (min)				
<10	0.02	(0.01-0.06)	20.1	(16-24)
10-19	0.01	(0.001-0.002)	18.8	(15.9-21.7)
20-29	0.06	(0.02-0.1)	14.9	(11.3-18.5)
30-39	3.4	(1.5-5.2)	14.4	(11.4-17.5)
45-59	3.0	(1.9-4.2)	61.6	(56.4-66.8)
≥60			63.7	(59.7-67.6)
Group B^***^				
Weekly frequency (days)				
Daily	1.8	(0.2-3.4)	3.7	(1.0-6.3)
5-6	2.8	(0.8-4.7)	5.9	(0.2-11.5)
3-4	6.1	(2.4-9.7)	4.2	(2.4-6.0)
1-2	89.3	(85.0-93.7)	86.2	(80.0-92.3)
Duration (min)				
<10	2.6	(0.2-5.0)	0.6	(0.1-1.2)
10-19	10.1	(4.7-15.6)	12.4	(6.3-18.5)
20-29	5.7	(2.8-8.7)	7.6	(4.4-10.7)
30-39	16.7	(10.9-22.4)	24.3	(17.7-30.7)
45-59	6.8	(3.9-9.7)	12.2	(6.6-17.7)
≥60	58.1	(50.2-65.7)	42.9	(35.4-50.4)

* Percentage weighted to adjust the Vigitel-2014 sample distribution to the population distribution projected for the 2014 year.

** Reach the WHO recommendations goals (2010) - Sum of active and very active.

*** Don't reach the WHO recommendations goals (2010) - Sum of inactive and insufficient active.

95% CI - 95% Confidence interval.

Conversely, most of the hypertensive patients in Group B presented weekly frequency of 1 to 2 days in a week, with 89.3% (95% CI: 85.0-93.7) among men and 86.2% (95% CI: 80.0-92.3) among women.

## Discussion

This cross-sectional study aimed at describing the daily PA profile of Brazilian adults with self-reported arterial hypertension in 2014 and at verifying if the weekly frequency of activities meets the WHO recommendations. Walking, soccer and water aerobics were the main modalities of exercise and sport practiced by hypertensive individuals. The weekly frequency of effort led 35% participants to achieve the recommended goal. The reduced weekly frequency of once to twice a week was highlighted as a barrier for hypertensive individuals and could not reach the recommended goal.

The increase in cases resulting from hypertension complications has reflected on the management of health system and increased costs for its control and treatment ^16^. Health surveillance measures, based on healthier habits adoption, are determinant to control this disease. In this perspective, the Brazilian Ministry of Health published, in 2014, the Strategies for the Care of People with Chronic Disease: systemic arterial hypertension. The main goal was guiding chronic patients to practice aerobic exercises with gradual overload within the individual limit. The recommended frequency was 3 to 5 times a week, at least 30 min a day, with activities lasting not less than 10 min. Anaerobic exercises were also recommended from 3 to 5 times a week for individuals with systolic and/or diastolic blood pressure below 160 or 105 mmHg, respectively.

The Ministry of Health, based on its guidelines, does not predict the anaerobic efforts duration and does not present the basis of studies that show the hypotensive effects of anaerobic exercise practice ^11^. The weekly frequency and the duration of effort are used to calculate weekly frequency and determine PA goal achievement.

In this study, effort duration was in agreement with most of international recommendations, which lasts 30 min or more. Investments should focus on encouraging increased regularity of activities. Many hypertensive patients, who did not reach the recommended goal, reported to take part of physical activities only once to twice a week. In general, PA level of hypertensive individuals was low and only 35% of them have reached this goal. However, the most worrying situation for hypertensive health was the complete absence of PA for 60% of them. Although there are some divergences concerning different PA recommendations, especially with regard to the ideal way of obtaining the minimum frequency, it is a consensus among the guidelines that the dose-response ratio is greater when inactive individuals incorporate some PA level than by increasing activity among the moderated active ones ^9^ ^,^ ^10^ ^,^ ^12^. Therefore, starting some PA practice seems to be, for most hypertensive patients, the most important step.

The obstacles to take part of PA may differ according to sex, city size, physical environment, mood, and health condition of an individual. According to the data found out by Andrade et al.^17^, the most frequent obstacles for both sexes in small cities are: a) lack of equipment; b) need for resting; c) lack of an appropriate place; d) lack of adequate weather; And e) lack of physical ability. In the largest cities, the most frequent problems are: a) lack of equipment; b) lack of time; c) lack of knowledge; d) fear of injury; And e) need of resting. The favorable physical environment tends to decrease the strength of these barriers and influence the behavior for PA participation ^18^. 

Thus, aesthetic and functional adaptations of a physical environment around residential areas, creation of safe environments on sidewalks and crossings, reduction of traffic speed, preventive policing, extending of opening hours on physical facilities and dissemination of correct places for PA may be appropriate as a facilitating strategy for hypertensive individuals to remain and become active ^19^ ^,^ ^20^.

The walking practice was preferred by almost 67% of hypertensive adults that were studied in this trial as the main modality of exercise or sport. This adherence by hypertensive patients was higher than the one observed for public in general (43%) ^9^. Thus, walking, classified as a moderate-intensity aerobic activity, was the one that contributed most to achieve this goal, in accordance with the WHO recommendations that recommends this kind of effort on a preferential basis ^12^.

Knowing the previous diagnosis of hypertension and obtaining information about PA benefits on this health disorder may reinforce the idea for hypertensive patients to be or remain physically active. Consequently, these actions may contribute indirectly to prevent disease complications or the emergence of comorbidities related to sedentary habits. Thus, it seems opportune to add intersectional public health efforts in order to encourage and adapt the participation of hypertensive patients on physical activities (PA).

This study has some restrictions that must be addressed. Vigitel data only included the main modality practiced during the last three months and does not allow capturing information from other complementary practices. The measurement of self-referenced survey effort does not provide the real level of an effort for each individual. Direct and more accurate decision-makings are expensive and logistically difficult in a large sample as in this study. Since PA is conceived by the population as a desirable practice, it should be considered the possibility of reluctance to be declared as inactive.

However, Monteiro *et al*.^21^, have evaluated PA reproducibility and validity as well as sedentary lifestyle indicators obtained by Vigitel with an 80% specificity index. They also concluded that both PA and sedentary indicators tend to be reproducible and accurate. Since the system does not aim at determining prevalence, but monitoring them, information concerning arterial hypertension detection has not been checked *in loco*. Another limitation refers to the use interviews by landlines (telephones), since it does not consider people who do not have landlines. The use of post-stratified weights minimizes possible differences between the total population and the studied one.

The strengths of this study include information on a large sample of Brazilian adults with hypertension and their participation in PA, detailing what kind of PA they practice, their weekly frequency and effort duration. This study provides relevant evidences, especially for health professionals and stakeholders interested in planning and promoting PA for hypertensive patients, in order to reinforce actions to encourage people to be more active, to reduce inequalities of access between sexes and age groups as well as to ensure suitable and safe public spaces in order to put in practice PA.

Primary Care services in a public and universal health system are also important to be highlighted as a feasible place that focuses on PA benefits, due to they are close and can easily get in touch with population, especially among the hypertensive ones.

It is concluded that, in Brazil, the number of inactive hypertensive people (60%) is significant, mainly among women. It was also recorded that hypertensive people seldom practice exercise in a week, thus, they did not reach the goal recommended by the WHO. Consequently, it is essential to understand how PA participation is inserted in different areas. There is also a requirement of health professionals and public policy managers to encourage inactive hypertensive patients to adopt active living habits as a priority due to their frequency in physical activities.
