# Supplementary material for: Daily physical activity of Brazilian carriers of arterial hypertension: a transversal analysis
Source: Colomb Med (Cali). 2017 Jun 30;48(2):82–7. (PMC5549888)
Supplement: Translation

# Translation: Actividad física diaria de los pacientes hipertensos de brasil: un análisis transversal

## Introducción

Se estima que en escala mundial hay un tercio de hipertensos no conscientes de su condición, aumentando el riesgo de infarto de miocardio, accidente cerebrovascular y enfermedad renal. En el mundo la hipertensión es responsable de alrededor 9.4 millones de muertes y 10% del costo de la salud. Según las estimaciones en el año 2025, la hipertensión alcanzará cerca de 1.6 millones de personas de ambos sexos ^1^. En Brasil, la hipertensión afecta al 30% de la población adulta, alcanzando 5% en niños y en adolescentes y 50% en los ancianos ^2^. De acuerdo con un estudio que incluyó 190 países entre 2001-2011, el Brasil tiene la sexta proporción más alta de la hipertensión en el mundo (552/100 mil habitantes) ^3^.

En este escenario, un aspecto positivo es la posibilidad de que la hipertensión arterial se puede controlar con el uso de medicamentos antihipertensivos y con la adopción de hábitos de vida saludables. El efecto antihipertensivo causado por la actividad física regular (AF) ha apoyado su práctica terapéutica como una medida complementaria. Un metaanálisis realizado por Fagard ^4^, mostró uma disminución significativa en la presión sistólica y diastólica (3.4 y 2.4 mmHg, respectivamente, *p* <0,001) causada por el entrenamiento aerobio realizado de 3 a 5 veces a la semana y 30 a 60 min de duración. Este resultado fue confirmado por otros estudios ^5^^-^^7^.

En Brasil, uu estudio basado en la monitorización de la hipertensión analizó el nivel de AF de 9,038 sujetos con hipertensión (60 años o más de edad), resultando en una alta prevalencia de AF insuficiente (88%; 95% CI 86-89) ^8^. Otro estudio representativo con la participación 11,453 hipertensos con 18 años o más de edad mostró 59.5% (IC 95%: 57.8-61.0) de inactivos, 10.1% (IC 95%: 8.8-12.2) de activos insuficientes y 30.4% ( IC 95%: 26.8-32.8) de activos suficientes (*p* = <0.001) ^9^.

Actualmente, varias organizaciones recomiendan AF para promover la salud de los hipertensos. A primera vista, parece que hay ambigüedad en la distribución del volumen de AF en la semana. Sin embargo, están de acuerdo con la necesidad de alcanzar una dosis mínima para dar efectos significativos la salud. Por ejemplo, el Colegio Americano de Medicina Deportiva recomienda ejercicio moderado de una duración de 20 a 40 min por día, de forma continua o fraccionada en tres días en la semana no consecutivos ^10^. El Ministerio de Salud brasileño recomienda AF 3 a 5 veces a la semana, por lo menos 30 min al día, con el grado de intensidad según la condición fisiológica del individuo ^11^. La Organización Mundial de la Salud (OMS), acompañado por el Departamento de Salud Nacional de Estados Unidos y apoyado por pruebas sólidas acerca de los beneficios de la AF para la salud de los hipertensos se producen independientemente del tipo y la intensidad del esfuerzo, recomienda la meta de 150 min por semana de actividad aeróbica de intensidad moderada o 75 min de intensidad vigorosa o una combinación equivalente de ambas, preferiblemente, pero no necesariamente, distribuidas en la semana, advirtiendo que el tipo y la cantidad de actividad deben ser determinados por la habilidad individual y limitaciones de la enfermedad ^12^^,^^13^.

En esta perspectiva, este trabajo tuvo como propósito describir el perfil de la AF de adultos brasileños portadores de hipertensión arterial que viven en las capitales de los estados de la Federación de Brasil y Distrito Federal y analizar si el volumen de AF esta de acuerdo con las recomendaciones de la OMS.

## Materiales y Métodos

Este es un estudio de tipo descriptivo y corte transversal con uso de datos secundarios del Sistema de Vigilancia de las Enfermedades Crónicas no Transmisibles y mediante Entrevistas Telefónicas (Vigitel), en Brasil, para el año 2014, cuya metodología se puede acceder a la publicación previa ^14^. Desde el año de 2006, las entrevistas del sistema se realizan anualmente mediante una entrevista telefónica con los adultos de edad superior a 18 años que viven en 26 capitales brasileñas y el Distrito Federal, cuyos hogares poseen una línea telefónica. Por razones estratégicas de monitoreo, algunas preguntas de la investigación pueden ser añadidas o eliminadas. Cuestiones relativas al compromiso en la AF no han cambiado con el tiempo. En el año 2014, 62,786 líneas telefônicas fueron elegibles, dando como resultado 40,853 entrevistas válidas, lo que indica una tasa media de 65.2% de éxito. La duración media de las entrevistas fue de 9.5 min. Las entrevistas se realizaron por médio de um cuestionario electrónico que cubrió las características demográficas, socioeconómicas y de comportamiento de los individuos y preguntas acerca de la práctica cotidiana de la AF. Para ajustar la población adulta de cada ciudad se le asignó un peso final para cada individuo entrevistado, denominado peso post-estratificado, con el objetivo de corregir la representación de los estratos sociodemográficos que podrían ocurrir debido a la no cobertura universal de red de línea fija ^15^.

### Variables

Este estudio consideró hipertenso todos los participantes que respondieron afirmativamente a la pregunta: ¿Alguna vez un médico le ha dicho que tiene la presión arterial alta? Para las estimaciones de nivel de AF considera la suma de la frecuencia semanal y duración de las actividades ejecutada durante el tiempo libre o ir al trabajo o estudio mediante a la preguntas: ¿En los últimos tres meses, se practica algún tipo de ejercicio o deportes físicos? ¿ Para ir a trabajo o estudio, hace algún camino a pie o en bicicleta?

La intensidad del esfuerzo para cada tipo de ejercicio o deporte siguió a la clasificación establecida por lo Vigitel - 2014. Así, caminata, caminata en la cinta, aeróbico acuático, gimnasia general, natación, ciclismo y voleibol fueron clasificados como prácticas de AF de intensidad moderada. Carrera, carrera en la cinta, fisicoculturismo, gimnasia aeróbica, artes marciales, fútbol, básquetbol y tenis fueron clasificados como prácticas intensidad de AF vigorosas.

La exposición diaria al ejercicio se determinó con las preguntas, "¿Cuál es la duración de su actividad?". La duración fue clasificada en seis grupos: menos de 10 min; 10 a 19 min; 20 a 29 min; 30 a 39 min; 45 a 59 min; igual o mayor que 60 min. Actividad que dura menos de 10 min no se consideró para fines del cálculo ^12^.

La frecuencia semanal de la AF se determinó por el número de días activos en la semana, mediante la pregunta: "¿cuántos días a la semana usted practica ejercicio físico o deporte?". Para el análisis, la frecuencia semanal se clasificó en cuatro grupos: a diario, de 5 a 6 días, 3 a 4 días y de 1 a 2 días a la semana.

El análisis de la participación en la AF consideró sólo los últimos tres meses anteriores a la entrevista. Originalmente, la encuesta del Vigitel separada AF en cuatro áreas: AF de recreo (tiempo libre); AF ocupacional (de trabajo); AF en la casa (limpieza doméstica); y AF en los desplazamientos de casa al trabajo o estudio (a pie o en bicicleta). En este estudio, sólo las información de recreo y el transporte activo se utilizaron para establecer el nivel de AF de los participantes. Las actividades en el ámbito doméstico y ocupacional, no se consideraron debido este tipo de interrogatorio no permitir cuantificar su duración y frecuencia semanal.

La división de las regiones del país atendió a la agrupación de unidades de la federación de Brasil. Actualmente, hay cinco regiones oficiales: la región Centro-Oeste con tres estados (Goiás, Mato Grosso y Mato Grosso do Sul) y el Distrito Federal; región Nordeste con nueve estados (Alagoas, Bahía, Ceará, Maranhão, Paraíba, Pernambuco, Piauí, Rio Grande do Norte y Sergipe); región Norte con siete estados (Acre, Amapá, Amazonas, Pará, Rondônia, Roraima y Tocantins); región Sudeste con cuatro estados (Espirito Santo, Minas Gerais, Río de Janeiro y Sao Paulo); región Sur con tres estados (Paraná, Rio Grande do Sul y Santa Catarina).

El nivel de la AF se determinó mediante los parámetros de la OMS, que considera suficiente la práctica de 150 min de AF moderada y 75 min de AF vigorosa por semana ^12^.

Inicialmente, los participantes fueron agrupados en cuatro categorías: 1) Inactivo: negó la práctica de AF de recreo y el transporte activo; 2) insuficiente activo: mantiene AF por debajo de la recomendación; 3) Activo: alcanza a la meta recomendada; 4) muy activo: superó la meta recomendada. Posteriormente, se realizo un segundo agrupación con dos categorías: Grupo A: se compone de la suma de los participantes activos y muy activos. Grupo B: se compone de la suma de participantes inactivos y activos suficientes.

La prevalencia de la AF de los hipertensos y sus respectivos intervalos de confianza de 95% se presentan según el nivel individual de la AF, por región y por tipo de ejercicio y/o deporte. Fueron descritas características de la AF de la población hipertensa comparándola con la población en general. Posibles diferencias estadisticas en la prevalencia de la AF de acuerdo con la presencia o no de la enfermedad se obtuvieron con la puebla de Chi cuadrado de Pearson (χ2). Los resultados con *p* inferior a 0.05 se consideraron significativos a nivel estadístico. Para el procesamiento de los datos y el análisis estadístico se utilizó el Stata^®^ 11.0 y 10.0 programas de Microsoft^®^ Excel.

### Aspectos éticos

Este estudio se realizó con datos secundarios colectados por el sistema Vigitel. El proyecto Vigitel fue aprobado por la Comisión Nacional de Ética en Investigación en Seres Humanos del Ministerio de Salud (Conep - Parecer 26/06/2013 355.590 - CAAE: 16202813.2.0000.0008). Se obtuvo el consentimiento informado por vía oral en el momento del contacto telefónico con los entrevistados y se aseguró el anonimato de los participantes.

## Resultados

Los datos analizados del Vigitel fueron del año 2014, con la participación de 40,853 entrevistados con edad media de 48.3 ± 17.6 años, compuesto por 15,521 hombres con la edad media de 45.9 ± 17.5 años y 25,332 mujeres con la edad media de 49.7 ± 17.5 años. La mayoría de la población se declaró blanco (44.9%) o moreno (40.9%). Solamente el 1.6% de los entrevistados se declararon indígenas y el 9.8% se declararon negros. Las regiones del país con el mayor número de estados tienen mayor participación, pero los pesos de post-estratificación equilibra la muestra a cada ciudad.

Fueron excluidos de este estudio 1,405 participantes (3.4%) qué no reportaron la AF sobre la frecuencia semanal o la duración. Por lo tanto, la base para el cálculo consideró 39,448 participantes. Entre los que confirmaron el diagnóstico de la hipertensión (23.5%; IC 95%: 21.6-25.4), hubo más mujeres (25.8%; IC 95%: 22.2-27.6) que hombres (20.3%; IC 95%: 18.4-22.9) (*p* = 0.001). La ciudad de Palmas presentó la menor prevalencia de hipertensión (15.2%) y Porto Alegre, la mayor (29.2%).

La distribución de la prevalencia de la hipertensión es desigual en cuanto a la escolaridad. Las personas más afectadas tenían ocho años de escolaridad (38.1%: IC 95%: 36.6-40.8); 19.3% (IC 95%: 17.5-21.7) a partir de 9 a 11 años y 14,6% (IC 95%: 11.9-16.6) tenían más de 12 años (*p* = 0,001). Mulatos tenían una menor prevalencia de hipertensión (4.7%) y los de origen asiático la mayor (10.4%) (*p*= 0.01). En ambos sexos, la prevalencia de hipertensión fue mayor con la edad avanzada. Ha caído desde 4.6% en las personas entre 18-24 años de edad, a 59.9% en las personas mayores de 65 años.

La Tabla 1 muestra la distribución de hipertensos de acuerdo con el nivel de la AF. Se nota que más de la mitad (59.5%) de las personas fueron inactivas y sólo el 34.4% disponían de un nivel suficiente de AF. La Tabla 2 muestra la prevalencia de la hipertensión y el porcentaje que alcanzaron la meta de AF recomendado por la OMS. Los hombres hipertensos más activos residían en la región Centro-Oeste (44.1%; IC 95%: 24.0-53.0) y los hombres en la región Sur fueron menos activos (36.6%; IC 95%: 26.0-46.0). Las mujeres hipertensas eran más activas en la región Centro-Oeste (36.6%; IC 95%: 28.0-44.0) y menos en el Norte (27.3%; IC 95%: 19.0-36.0).

**Tabla 1:** Distribución proporcional* de hipertensos de edad ≥ 18 años, por sexo, de acuerdo com el nivel de la AF determinado por las recomendaciones de la OMS^1^ en las capitales brasileñas y en el Distrito Federal, Brasil, 2014.

	Total		Hombres		Mujeres	
Nível de AF	%^#^	IC 95%	%^#^	IC 95%	%^#^	IC 95%
Inactivo	59.3	55.8 - 59.2	54.3	50.6 - 56.4	64.3	62.1 - 6.9
Activo Insuficiente	10.7	8.8 -12.2	13.5	11.3 - 14.3	7.8	6.6 - 9.1
Activo	8.8	7.1 - 9.5	7.9	6.0 - 9.3	7.8	6.3 - 8.6
Muy activo	21.2	19.7 - 23.3	24.3	22.8 - 6.3	20.1	18.9 - 2.9
Grupo A^**^	34.4	31.4 - 38.1	39.8	35.3 -43.9	31.3	28.6 - 5.9
Grupo B^***^	65.6	61.9 - 69.4	60.2	56.1 -65.2	68.7	64.0 - 62.1

* Porcentaje ponderada para ajustar la distribución de la distribución de la muestra Vigitel-2014

^**^ Alcanza las recomendaciones de la OMS 2010 - Suma de los activos y muy activo.

*** No alcanza las recomendaciones de la OMS 2010 - Suma de los inactivos e activos insuficientes.

^#^Test Chi cuadrado de Pearson <0.005.

**Tabla 2:** Porcentaje^*^ de personas hipertensas de edad ≥18 años que alcanzó las recomendaciones de AF de la OMS^1^-2010, por sexo y regiones de Brasil, 2014.

Regiones	Meta alcanzada			χ^2^
Pre^**+**^ %	Hombres IC 95%	Mujeres IC 95%		
Norte	19.4	36.6 (26.0-46.0)	27.3 (19.0-36.0)	<0.001
Nordeste	24.3	41.9 (31.0-48.1)	30.7 (22.0-38.0)	<0.001
Centro Oeste	24.7	44.1 (24.0-53.0)	36.6 (28.0-44.0)	<0.001
Sudeste	26.1	36.7 (26.0-46.0)	30.2 (26.0-38.0)	<0.001
Sur	25.0	38.4 (29.0-49.0)	36.1 (29.0-43.5)	<0.001

* Porcentaje ponderada para ajustar la distribución de la distribución de la muestra Vigitel-2014.

+ Prev = prevalencia de la hipertensión repostada.

IC 95% - Intervalo de Confianza del 95%.

X2 Test Chi cuadrado de Pearson.

La Tabla 3 muestra la forma principal de ejercicio o deporte practicado por los hipertensos. La caminata fue la principal actividad practicada por los integrantes del Grupo A (67.9%; IC 95%: 64.8-70.8) en comparación con el Grupo B (44.8%; IC 95%: 39.9-49.7). La preferencia por el fútbol fue predominantemente masculina, especialmente para los integrantes del Grupo B (41.6%; IC 95%: 34.1-49.1). El conjunto de los practicantes de los deportes en canchas deportivas contribuyó sólo com el 1% de todas las opciones.

**Tabla 3:** Porcentaje de la población adulta con hipertensión arterial*, el nivel de AF por sexo y tipo de AF en las capitales de los estados brasileños y en el Distrito Federal, Brasil, 2014.

Hipertensión	Grupo A^**^						Grupo B^***^					
Modalidades	Total %	IC 95%	Hombres %	IC 95%	Mujeres %	IC 95%	Total%	IC 95%	Hombres %	IC 95%	Mujeres %	IC 95%
1= caminata	67.9	64.8-70.8	60.4	55.3-65.4	73.7	70.4-7.5	44.8	39.9-49.7	31.2	25.1-37.3	59.9	53.0-66.7
2= caminata - cinta	1.9	1.2-2.6	2.6	1.4-3.8	1.4	0.6-2.2	1.6	0.5-2.7	1.8	0.01-3.5	1.5	0,2-2.8
3= carrera	2.7	1.7-3.7	3.9	1.9-5.8	1.8	0.9-2.6	2.0	0.8-3.1	2.3	0.6-4.0	1.5	0.08-3,2
4= carrera - cinta	0.3	0.05-0.6	0.6	0.01-1.2	0.1	0.04-1.3	0,4	0.1-0.8	0.6	0.3-1.6	0.04	0.05-0.1
5= musculación	6.3	4.6-7.8	7,5	4.8-10.2	5,2	3.4-7.1	2.9	0.7-5.1	3.7	0.1-7.5	2.0	0,2-3.8
6= gimnasia aeróbica	2.0	1.0-3.0	0.7	0.02-1.4	3.0	1.3-4.7	1.3	0.6-1.9	0.6	0.05-1.3	1.9	0.8-3.1
7= hidrogimnasia	3.7	2.5-4.8	1.1	0.3-2.1	5.7	3.8-7.6	5.4	3.1-7.7	1.5	0.2-2.8	9.8	5.3-14.3
8= gimnasia	2.6	1.8-3.3	1.3	0.5-2.0	3.6	2.3-4.9	4.6	3.0-6.2	2.7	0.9-4.5	6.7	4.1-9.4
9= natación	0.9	0.4-1.4	1.1	0.3-1.9	0.7	0.005-1.4	2.3	1.0-3.6	2.6	0.6-4.5	2.0	0.3-3.8
10= artes marciales	0.3	0.02-0.5	0.6	0.03-1.2	0.2	0.001-0.05	0.3	0.03-0.6	0.3	0.1-0.8	0.2	0.2-0.6
11= ciclismo	3.2	2.4-4.1	4.4	3.0-5.8	2.3	1.2-3.3	5.0	2.4-7.7	5.4	2.4-8.4	5.0	0.3-9.1
12= fútbol	4.7	3.1-6.3	10.5	7.0-13.9	0.05	0.004-0.1	22.2	17.4-27.0	41.6	34.1-49.1	0.6	0.2-1.2
13= básquetbol	0.1	0.01-0.3	0.2	0.03-0.5	0.01	0.007-0.02	0.005	0.005-0.02	0.01	0.001-0.03	**-**	**-**
14= voleibol	0.2	0.05-0.5	0.4	0.02-1.1	0.06	0.005-0.01	1.0	0.2-2.3	0.6	0.07-1.0	1.5	1.1-4.1
15= tenis	0.1	0.01-0.02	0.3	0.01-0.5	0.08	0.01-0.1	0.3	0.04-0.6	0.4	0.02-0.8	0.2	0.1-0.5
16= Otros	3.1	1.8-4.4	4.4	1.9-6.8	2.1	0.8-3.4	5.9	3.8-8.0	l4.7	1.5-7.9	7.2	5.6-10.0

* Porcentaje ponderada para ajustar la distribución de la distribución de la muestra Vigitel-2014.

* * Alcanza las recomendaciones de la OMS 2010 - Suma de los activos y muy activo.

*** No alcanza las recomendaciones de la OMS 2010 - Suma de los inactivos e activos insuficientes.

IC 95% - Intervalo de Confianza del 95%.

La Tabla 4 muestra la distribución de la frecuencia semanal y la duración de las AF. La mayoría de los hombres (39.5%; IC 95%: 34.4-44.5) y las mujeres (43.1%; IC 95%: 38.9-47.3) del Grupo A participaron en la AF con 3-4 días a la semana. Sólo el 5.5% (IC 95%: 3.2-7.7) para los hombres y el 1.1% (IC 95%: 0.2-1.9) de las mujeres participaron con 1-2 días a la semana. Más de la mitad de los hipertensos practicaron AF durante 60 min al día y aproximadamente el 90% practicaron durante 30 min o más. Por el contrario, la mayoría de los hipertensos del Grupo B tuvieron la AF durante 1 a 2 días a la semana, 89.3% (IC 95% 85.0-93.7) entre los hombres y 86.2% (IC 95%: 80.0-92.3) entre las mujeres.

**Tabla 4:** Porcentaje* de la población adulta con hipertensión arterial, nivel de AF por sexo, según la frecuencia semanal y duración de la actividad en las capitales de los estados brasileños y en el Distrito Federal, Brasil, 2014.

Grupo A^**^	Hombres (%)	IC 95%	Mujeres (%)	IC 95%
Frecuencia semanal				
Diario	29.8	24.2-35.4	22.7	18.8-26.6
5-6	25.2	20.4-30.0	33.1	29.1-37.1
3-4	39.5	34.4-44.5	43.1	38.9-47.3
1-2	5.5	3.2-7.7	1.1	0.2-1.9
Duración (min)				
<10	0.02	0.01-0.06	20.1	16-24
10-19	0.01	0.001-0.002	18.8	15.9-21.7
20-29	0.06	0.02-0.1	14.9	11.3-18.5
30-39	3.4	1.5-5.2	14.4	11.4-17.5
45-59	3.0	1.9-4.2	61.6	56.4-66.8
≥60			63.7	59.7-67.6
**Grupo B** ^***^				
Frecuencia semanal				
Diario	1.8	0.2-3.4	3.7	1.0-6.3
5-6	2.8	0.8-4.7	5.9	0.2-11.5
3-4	6.1	2.4-9.7	4.2	2.4-6.0
1-2	89.3	85.0-93.7	86.2	80.0-92.3
Duración (min)				
<10	2.6	0.2-5.0	0.6	0.1-1.2
10-19	10.1	4.7-15.6	12.4	6.3-18.5
20-29	5.7	2.8-8.7	7.6	4.4-10.7
30-39	16.7	10.9-22.4	24.3	17.7-30.7
45-59	6.8	3.9-9.7	12.2	6.6-17.7
≥60	58.1	50.2-65.7	42.9	35.4-50.4

* Porcentaje ponderado para ajustar la distribución de la distribución de la muestra Vigitel-2014.

** Alcanza las recomendaciones de la OMS 2010 - Suma de activos y muy activos.

*** No alcanza las recomendaciones de la OMS 2010 - Suma de inactivos y activos insuficientes.

IC 95% - Intervalo de Confianza del 95%.

## Discusión

El objetivo de este estudio transversal fue describir el perfil de la AF de los adultos brasileños con hipertensión arterial en 2014 y comprobar si la cantidad semanal de la AF cumple con las recomendaciones de la OMS. La caminata, el fútbol y la gimnasia fueron las principales formas de ejercicio y deporte practicado por hipertensos. La AF semanal conllevó a que el 35% de los participantes lograron el objetivo recomendado. La frecuencia semanal de AF durante 1-2 veces a la semana (reducida) se presentó como una barrera para que este grupo de hipertensos lograra alcanzará el nível recomendado.

El aumento de los casos debido a complicaciones de la hipertensión afecta a la gestión del sistema de salud con aumento de los costes para su control y tratamiento ^16^. Medidas de vigilancia de la salud basado en la adopción de hábitos de vida más saludables son esenciales para controlar la enfermedad. En esta perspectiva, el Ministerio de Salud de Brasil publicó en el 2014, las Estrategias de Atención a las Personas con Enfermedades Crónicas: Hipertensión Arterial Sistémica, guiando a los enfermos crónicos para practicar ejercicios aerobicos con sobrecarga progresiva dentro del límite individual. La frecuencia semanal de AF recomendada fue al menos de 3 a 5 veces por semana y 30 min por día con duración de la actividad no menor de 10 min. Se recomiendan ejercicios anaerobicos con frecuencia de 3 a 5 veces a la semana para los pacientes con presión arterial sistólica y diastólica menor de 160 y 105 mmHg, respectivamente. El Ministerio de Salud, en sus directrices, no prevê la duración de los esfuerzos anaerobios y no mostró la base de estudios que motivaron la decisión ^11^.

La frecuencia semanal y la duración de la AF determinan el volumen semanal para estimar el alcance de la meta. En este estudio, la duración de la AF se presentó en la mayoría de acuerdo a las recomendaciones, es decir, tiene una duración de 30 min o más. Las políticas deben centrarse en fomentar el aumento y la regularidad de las actividades. Muchos hipertensos que no alcanzaron la meta participan en la AF sólo durante 1-2 veces a la semana. Generalmente, el nivel de AF de los hipertensos fue bajo, sólo el 35% alcanzaron la meta. Sin embargo, la situación más preocupante encontrada para la salud de los hipertensos fue la ausencia completa de AF para el 60% de ellos. Aunque hay algunas diferencias entre los diferentes recomendaciones de AF, en particular en relación con la forma ideal para obtener el volumen mínimo, es de consenso que la relación de la dosis-respuesta es más pronunciada cuando los inactivos incorporan un cierto grado de AF, mas que que por el incremento de la actividad entre los moderadamente activos ^9^^,^^10^^,^^12^. En este sentido, comenzar a practicar alguna AF parece ser, para la mayoría de los hipertensos, el paso más importante.

Las barreras a la participación en la AF pueden ser diferentes según el sexo, tamaño de la ciudad, el entorno físico, la disposición y el estado de salud del individuo. De acuerdo con los datos presentados por Andrade *et al.*^17^, las barreras más frecuentes para ambos sexos en ciudades pequeñas son: a) la falta de equipos; b) la necesidad de descanso; c) la falta de lugares apropiados; d) la falta de um clima adecuado; e) la falta de capacidad física, mientras en las ciudades grandes las barreras más comunes son: a) la falta de equipo; b) la falta de tiempo; c) la falta de conocimiento de sitios apropiados; d) miedo de uma lesión; y e) la necesidad de descansar. El entorno físico favorable tiende a disminuir la magnitud de estas barreras e influyen en el comportamiento de la participación en la AF ^18^. Por lo tanto, la adecuación estética y funcional del entorno físico en la zona de residencia, la creación de un entorno seguro en las aceras y cruces en las calles, la reducción de la velocidad del tráfico, la policía preventiva, la ampliación de las horas de funcionamiento de las instalaciones físicas y difusión de sitios adecuados para AF, pueden resultar oportuno como una estrategia facilitadora para que los hipertensos se mantengan o si conviertan en activos ^19^^,^^20^.

La práctica de caminata se alcanzó em cerca del 67% de los hipertensos como la principal forma de AF practicada. Esta adhesión por los hipertensos fue mayor que la observada para el público en general (43%) ^9^. Así, la caminata es la actividad que más contribuyó a la consecución de la meta, alineándose con las recomendaciones de la OMS ^12^.

El conocimiento del diagnóstico previo de hipertensión y la información sobre los beneficios de la AF sobre la enfermedad puede reforzar la idea en los hipertensos de mantenerse activos, contribuyendo indirectamente a la protección de las complicaciones de la enfermedad o la protección de enfermedades asociadas con hábitos sedentarios. Por lo tanto, vale la pena añadir los esfuerzos de los programas de salud pública intersectoriales para fomentar la participación y la de los hipertensos en AF.

Este estudio tiene algunas limitaciones que deben ser abordadas. La encuesta del Vigitel sólo incluye datos acerca de lo principal deporte practicado en los últimos tres meses y no permite capturar información de otras prácticas complementarias. La medida indirecta de la AF no permite comprobar el nivel real de esfuerzo de cada individuo. Las mediciones directas, más precisas, son costosas y logísticamente difíciles en muestras grandes como es el caso de este estudio.

La AF está diseñada para la población como una práctica deseable, así se debe considerar la posibilidad de la renuencia de la inactividad física. Sin embargo, Monteiro et al. ^21^, evaluaron la fiabilidad y validez de los indicadores de AF obtenidos por el Vigitel y obtuvieron um índice de especificidad el 80%, concluyendo que la AF tienden a ser altamente reproducible y precisa. Las informaciones sobre la presencia de hipertensión no se verificó *in situ*. Otra limitación se refiere a la utilización de las entrevistas telefónicas fijas, ya que no tienen en cuenta las personas que no tienen teléfono fijo. El uso de pesos post-estratificado minimiza la diferencia potencial entre la población total y población del estudio.

Los puntos fuertes de este estudio incluyen el gran tamaño de la muestra de los adultos brasileños con hipertensión y su participación en la AF, detallando el tipo de AF practicado, su frecuencia semanal y duración del esfuerzo. El estudio proporciona evidencias pertinentes, en particular para los profesionales de la salud y agentes interesados en la planificación y la promoción de la AF en hipertensos, para intensificar las medidas, para animar a las personas a ser más activas, reducir las desigualdades en el acceso, entre los sexos y grupos de edad y asegurar espacios públicos adecuados y seguros para la AF. Igualmente importante a destacar es la importancia de los servicios de atención primaria de salud en un sistema de salud pública y universal, como un lugar apropiado para asesorar sobre los beneficios de la AF, dada su proximidad y su influencia en la población general, especialmente entre los hipertensos.

Concluimos que, en Brasil, los hipertensos inactivos representan a la mayoría (60%), especialmente entre las mujeres. La frecuencia de AF semanal es reducida y es la característica más llamativa de los pacientes hipertensos que no alcanzan la meta propuesta por la OMS. Por lo tanto, es importante entender cómo la participación en la AF está incluida en los distintos segmentos de la población, lo que requiere de los profesionales de la salud y de los políticos, la tarea de fomentar hábitos regulares de AF entre los hipertensos inactivos.
